# Implementation of Lean Management in a Multi-Specialist Hospital in Poland and the Analysis of Waste

**DOI:** 10.3390/ijerph19020800

**Published:** 2022-01-12

**Authors:** Agnieszka Zdęba-Mozoła, Anna Rybarczyk-Szwajkowska, Tomasz Czapla, Michał Marczak, Remigiusz Kozłowski

**Affiliations:** 1 Department of Management and Logistics in Healthcare, Medical University of Lodz, 6 Lindleya Street, 90-131 Lodz, Poland; anna.rybarczyk@umed.lodz.pl (A.R.-S.); michal.marczak@umed.lodz.pl (M.M.); 2 Department of Management, Faculty of Management, University of Lodz, 90-237 Lodz, Poland; t_czapla@uni.lodz.pl; 3 Centre for Security Technologies in Logistics, Faculty of Management, University of Lodz, 90-237 Lodz, Poland; remigiusz.kozlowski@wz.uni.lodz.pl

**Keywords:** lean management, lean healthcare, 5Why, value-stream mapping, waste

## Abstract

At the beginning of the 21st century, Lean Management (LM) tools were introduced into the healthcare sector around the world. In Poland, there are still few LM implementations, and they are not of a comprehensive nature. The aim of this article is to present the application of the LM concept in a hospital in Poland as a tool for the identification and analysis of waste and its impact on the process of organizing the provision of medical services on the example of improvements in the process of patient admission. In the period from 1 July 2019 to 31 December 2019, a project of LM implementation was carried out at the Provincial Specialist Hospital in Wroclaw. The project was based on the method of value-stream mapping and 5Why. Standardized interviews (before and after the project) were conducted with people from the hospital management and middle-level managers. The implementation of LM tools resulted in the identification of a number of wastes, which have been divided into groups. The most important waste was paper medical documentation. Its change to an electronic form allowed for a better use of human capital resources; savings included 2.3 nursing positions and 1.09 medical staff positions.

## 1. Introduction

Lean Management (LM) was applied in the healthcare sector at the beginning of the 21st century. The main purpose of using Lean Management tools in this sector was to improve the quality of services provided to patients, to shorten the length of hospital stay, and to minimize the frequency of medical errors. It has therefore become necessary to implement solutions that improve efficiency, reduce cost, and engage employees to introduce an innovative approach in the organization [1].

The concept of Lean Management was developed and implemented in Toyota after World War II. The solutions introduced there made it possible to improve productivity, quality, and efficiency [2]. M. Graban defined a lean system as “a set of tools and a management system, a method of continuous improvement and employee involvement, a way for solving problems that are relevant to leaders and all levels of the organization” [3]. The overarching goal of Lean Management is to achieve the lowest cost, while maintaining the highest quality and in the shortest time [4]. The main task of LM is to “slim down” the organization from unnecessary procedures, activities, and inefficiencies by eliminating waste (jap. Muda). Identification of wastes and their subsequent removal is supposed to improve the processes occurring in the organization while ensuring high quality of manufactured products or services. An important element of the concept is the assumption of continuous improvement (kaizen), referring to continuous work on improving all elements of the process [5,6].

The first mentions of Lean Management solutions used in healthcare units can be found in publications from 1995 [7] and 1996 [8]. However, these were not structured activities, but merely descriptions of using individual methods (e.g., just-in-time). Pilot implementations in hospitals in the USA and Great Britain (in 2000 and 2002, respectively) [9] were aimed at improving the flow of patients and brought positive observations both in medical and non-medical areas [10]. The best known are the implementations at Virginia Mason Medical Centre in Seattle, Flinders in Australia, and the Royal Bolton NHS Foundation Trust in Farnworth (UK) [11].

The key principles of Lean Management primarily relate to two elements: values and the pursuit of excellence (by eliminating waste). Value is understood both in relation to the organization and to the client (patient) [12]. All activities undertaken in enterprises should aim at generating value [13]. Waste, on the other hand, is any action or part of a process that does not create value. There are seven types of waste in LM: transport, inventory, motion, waiting, overproduction, excessive processing, and defects [14]. In the healthcare sector, these are very visible, e.g., in the form of excessive movement of patients and staff, delays in deliveries, or ineffective management of resources [15].

The implementation of Lean Management projects in healthcare faces a number of barriers. The main obstacles are human barriers caused by the reluctance of healthcare unit employees and the lack of faith in the possibility of applying solutions [16] that have proved to be successful in production [17]. However, implementing lean thinking in an enterprise requires understanding the basic principles of Lean Management and adapting them to the specificity of a given unit, with particular emphasis on the diversity of departments [9,18]. Qualified experts [19], who have extensive knowledge of Lean Management implementation and are eager to get to know the hospital and familiarize themselves with its structure, organizational culture, the processes inside, and the staff are of key importance [20]. There is a view in the literature that LM can be used in any process in a healthcare unit, bringing measurable benefits, such as reducing inventories, shortening the duration of activities and processes, improving the quality of services, and increasing the satisfaction of both patients and employees [15,21].

**Figure 1:**
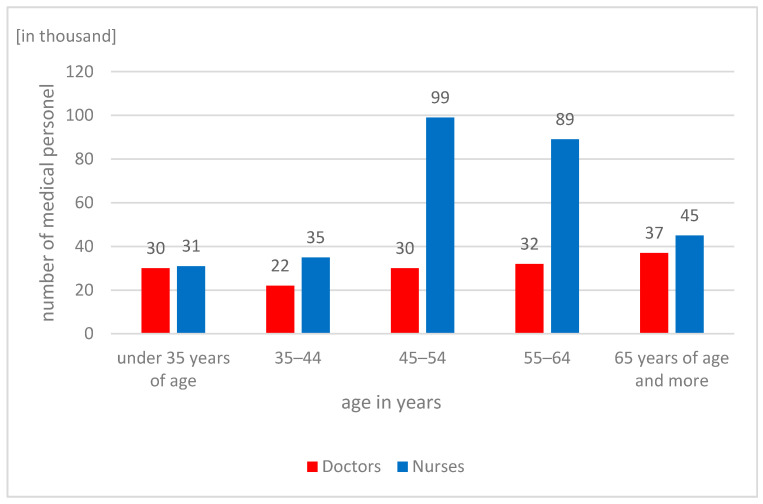
Medical and nursing staff divided into age groups. Status for 2019. Source: Own study based on the data of the Central Statistical Office.

Currently, Lean Management solutions are being used for the first time in Polish hospitals and outpatient clinics. One important project in the Polish healthcare system is the project of the National Centre for Research and Development, carried out by the Polish Society of Health Economics, and its partners, the aim of which is to develop national standards for Value-Stream Mapping (VSM) that can be used in various medical entities dealing with the treatment of patients with stroke [22]. However, these implementations are still few, and only single units show interest in the new methods [5]. The reasons why Polish hospitals use manufacturing industry solutions are the growing operating costs, aging medical personnel, and the constantly deteriorating healthcare situation. In the Euro Health Consumer Index report, in 2018, Poland was ranked 32nd out of 35 countries in Europe, achieving one of the worst results in all areas, especially in terms of patient rights, information, availability, and range and scope of operations [23]. The data of the Central Statistical Office show, in 2019, the tendency of persistent negative changes in the age structure of medical workers. The highest increase in the number of people authorized to practice medical profession was recorded in the oldest age group—65 and over. In 2019, the share of doctors in this group among all medical practitioners and dentists was 24.5%. Moreover, no changes were noted in the age group of 35–44 years. In 2017–2019, the size of this group remained the same, with the lowest level among all other groups (the share of this group among all physicians was 14.6%). Similar trends also persist among the nursing staff. In 2019, the largest number of nurses was in the age group of 45–54 years of age—99,000, while the least numerous group were nurses under 35 years of age. In the group of 35–44 years of age, for 8 years, a constant decrease has been observed in the number of people, with a constant annual increase in the group of 65 years and more. This trend confirms the aging of medical personnel in Poland, which is becoming a huge challenge for hospital managers [24]. A distribution of medical and nursing staff in individual age groups is presented in Figure 1.

Statistical analyzes of the Central Statistical Office also show slight changes in the increase of specialist doctors in Poland per ten thousand people. Compared to the data for 2019 and 2010, the number of medical personnel only slightly increased in the areas of radiodiagnostics, psychiatry, anesthesiology and intensive care, family medicine, surgery, and cardiology. In the other specialties analyzed by the Central Statistical Office, this number remained at the same level, while it decreased in the area of internal medicine [24].

**Figure 2:**
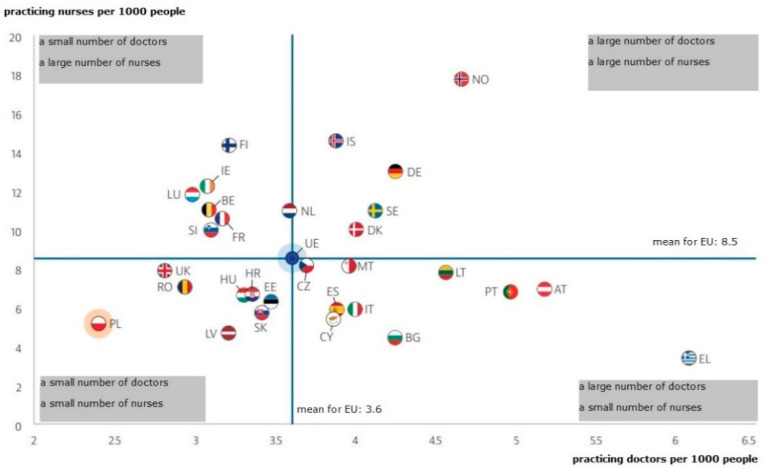
The number of doctors and nurses per one thousand inhabitants in individual EU countries. Note: for Portugal and Greece, the data refer to all licensed doctors, resulting in a significant overestimation of the number of practicing doctors (e.g., by around 30% for Portugal). For Austria and Greece, the number of nurses is underestimated as it only includes nurses working in hospitals. Source: OECD/European Observatory on Health Systems and Policies (2019), Poland: Country Health Profile 2019, State of Health in the EU, OECD Publishing, Paris/European Observatory on Health Systems and Policies, Brussels.

Poland is the country with the last position in the European Union in terms of the number of doctors and nurses per one thousand inhabitants, as evidenced by the data presented by the OECD and presented in Figure 2.

Such a difficult staffing situation in Polish hospitals implies the need to improve the efficiency of processes, their improvement, and elimination of unnecessary activities [20]. Moreover, units of the healthcare sector operate in conditions of constantly increasing demand for medical services; this is especially due to greater patient awareness and the aging of society [25]. Patients’ needs grow, and thus so does the necessity to provide them with satisfaction with treatment and services [26].

The aim of this article is to present the application of the LM concept in a hospital in Poland as a tool for the identification and analysis of waste and its impact on the process of organizing the provision of medical services on the example of improvements in the process of patient admission.

## 2. Materials and Methods

### 2.1. Overview of the Background of the Project

In 2019, a project was conducted to identify wastage using Lean Management tools. The project was implemented at the J. Gromkowski Provincial Specialist Hospital in Wroclaw.

**Figure 3:**
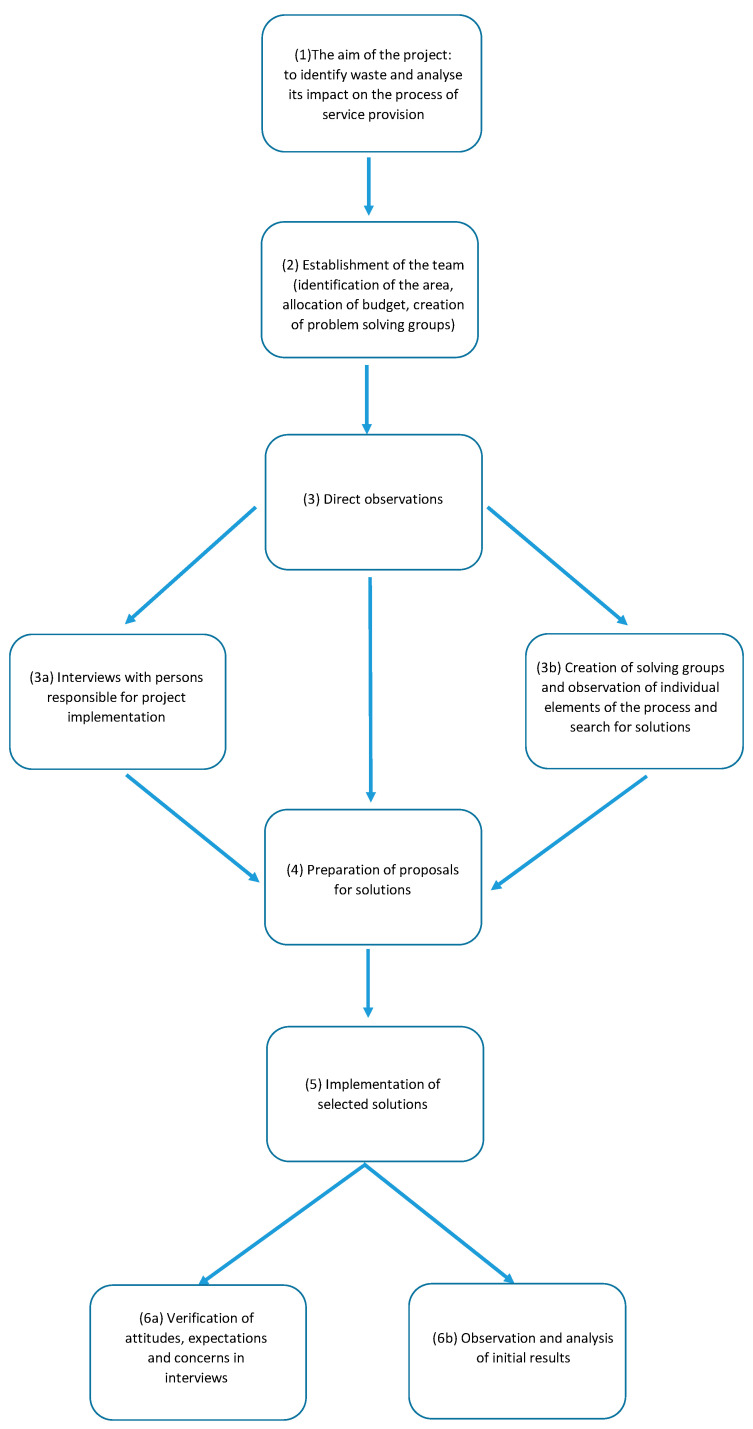
Scheme of the research project implementation process. Source: Own study.

Subsequent stages of the project are shown in Figure 3.

The project started on 1 July 2019 and lasted until 31 December 2019. The first step was to establish the project aim at the management level (1), followed by the appointment of a team (2). The team included: Deputy Director for Finance, Heads of Departments and Ward Matrons of Internal Medicine Department and Department of Gastroenterology, Manager of the Organization and Supervision Department, Head of the Human Resource Management Department, and external experts. The task of the team was to prepare the project budget, obtain external funds for its implementation, prepare and organize training courses for employees, set the project schedule, and then supervise its implementation. The team also identified the area that was designated to participate in the project. These included two internal medicine wards, a gastroenterology ward, and an admission room and laboratory. The departments had a total of 137 hospital beds. A total of 6162 patients were admitted to the departments in 2019. The revenues of the departments amounted to a total of EUR 5,334,031 (PLN 24,472,532.00), while costs amounted to EUR 6,182,530 (PLN 28,365,447.00), which means the loss of EUR 848,499 (PLN 3,892,913.00) in a year. In 2017 and 2018, the loss amounted to EUR 841,982 (PLN 3,863,015.00) and EUR 829,905 (PLN 3,807,604.00), respectively. The systematically deteriorating financial situation of the units and growing pressure to increase the effectiveness of the treatment were the direct reasons why the management of the hospital took steps to make changes in the organization of the work. However, it was not only the financial situation that convinced the hospital management that organization of the work of the departments required improvement.

In the subsequent stages, direct observations were made (3), interviews with managers responsible for project implementation were conducted (3a), and working groups (named groups solving) were formed. To this groups were appointed employees from the units where the project was implemented, and they, through their involvement in its implementation, became leaders of particular stages (3b). This was followed by meetings in working groups. Their task was to identify the most important areas for improvement, which were then passed on to members of the core team. Their task was then to verify the problems reported within the solving groups and the proposed solutions (4).

The next step was a formal implementation of solutions—changing the internal documentation, changing the organizational structure, including funds in the budget for the implementation of new ideas, and making changes in the purchase or investment plans (5).

The final stage was to verify the attitudes of managers responsible for the project—the extent to which their expectations and concerns were met (6a)—and to observe and analyze the initial results of the implemented solutions (6b).

The budget of the project was EUR 50,130 (PLN 230,00.00). As part of the project budget, the hospital financed the work of consultants from an external company and the work of hospital employees.

### 2.2. Characteristics of the LM Tools Used in the Project

The principles of Lean Management involve five main areas, and the project uses four of them directly related to the process:

1. Identifying the value generated by each process for the customer (external or internal). From the patient’s perspective, this is related to the process of admission and entire treatment. Lean refers to the need to design the diagram in a way that optimizes and eliminates unnecessary movements and activities.
2. Mapping the value stream of each service, which allows one to capture places that do not bring value and generate waste.
3. Improving the flow, where it is necessary to remove any barriers that hinder or delay the process.
4. Striving for perfection (Japanese kaizen) [15].

The fifth area—the pull system—refers to the time of delivery of the service product to the customer [27]. Delivery should take place when there is a need for a given product or service [28]. This area was not covered as the project did not include inventory management or verification of the timing of service delivery in line with patient needs [29]. However, the principles of the pull system relating to replenishment of resources after their use have been used in the implementation of electronic documentation.

In accordance with the principles of Lean Management, the work on the project took place in a place where the value was created (Japanese: gemba walk). The observations began in the place where the patient had direct contact with the hospital, i.e., in the admission room. The literature encounters the view that entering gemba and direct observation of the process allow for the best analysis of the value stream flow [12]. According to J. Womack [27], this brings measurable benefits. Working in the place where the service is produced enables managers to change the perspective and view the process exactly where it occurs. It improves the efficiency of the process and coaching of line managers. Communication, which is one of the basic elements of the effective implementation of Lean Management tools, is an important determinant of the project effectiveness [18]. The first part of the project involved a detailed analysis of the process of admitting a patient to hospital, which is a very important activities and one of the main processes in the unit. For this purpose, the trainers spent 14 days in all organizational units responsible for its implementation, i.e., admission room and departments, they participated in meetings of teams working in the departments, observed the preparation of documentation and ordering examinations, and analyzed the methods of in-hospital communication. Their main task was to prepare a map of the entire process, along with a report on identified problems/waste. Thanks to the use of the 5Why method (repeating the questions starting with the word “why” until the root cause of the problem was discovered) [9], in the first stage of the project, they not only made observations of the activities performed by the staff, but also asked questions in order to specify the elements of the process, understand their significance, or, at an early stage, identify problems and threats.

Subsequently, in the departments, the activities performed at individual positions were verified through the process of direct observations. The information was gathered from the people directly involved in particular tasks in individual positions. Free interviews focused on the possibilities of improving work. The trainers worked directly with the staff in the appropriate departments and recorded the activities performed by doctors, nurses, and administrative staff. The elements of a given process were noted in the cycle time measurement card, and then the notes were used to describe the entire process along with the characteristics of identified or reported problems and deficits.

The joint work resulted in the creation of a value stream map—a diagram of the process of patient admission and treatment. Mapping is a tool that allows for the recognition and visual presentation of individual elements of the process in order to optimize it, improve efficiency, eliminate problems, and improve the flow of value [30]. Therefore, the project inventoried the basic activities performed by medical and administrative staff in the process of admitting a patient to the hospital and transferring him/her to a department. Next, the stages of the process were carefully monitored in order to identify waste.

In the process of waste identification, working solving groups used A3 reports (Supplementary File S1). They consisted of the following elements:

1. Problem definition;
2. Determining the current state (along with a description of the most important problems and their dimensioning);
3. Defining the goal (and the main indicators of its implementation);
4. Analysis of the causes and steps necessary to achieve the target state;
5. Defining remedial measures, enabling the implementation of the next stages of the project;
6. Creating the work plan—persons responsible, tasks, schedule.

### 2.3. Main Findings of the Working Groups in the Project

#### 2.3.1. Patient Flow Analysis, Scope, and Method of Data Collection and Processing

**Figure 4:**
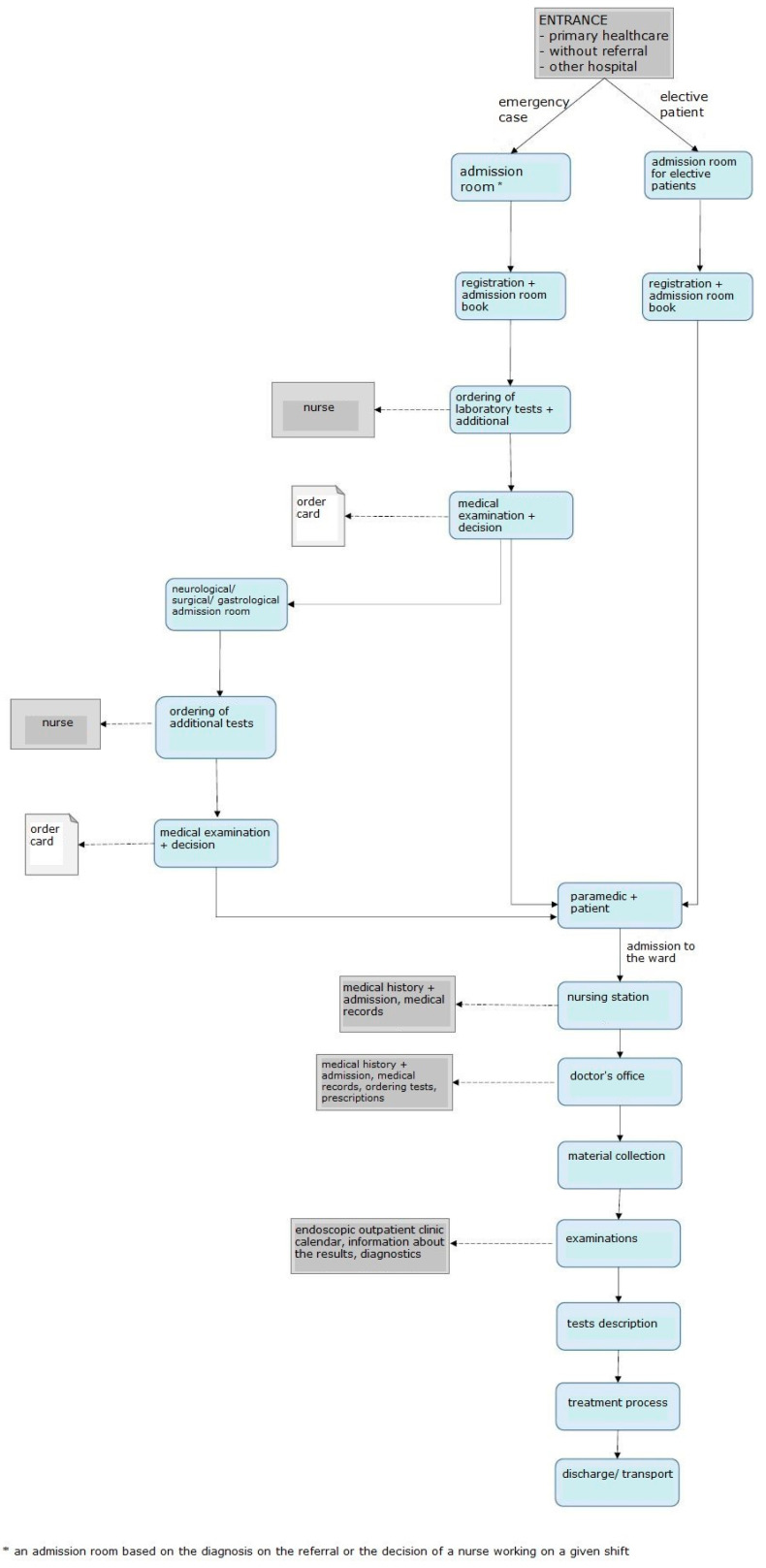
Diagram showing the process of admitting and treating a patient before the introduction of LM. Source: Own study.

The first problem identified in the admission room was excessive patient and emergency room staff traffic. Therefore, the processes related to the admission of a patient to the admission room, transfer to the ward, and discharge were analyzed and mapped. The resultant value stream map is shown in Figure 4.

**Figure 5:**
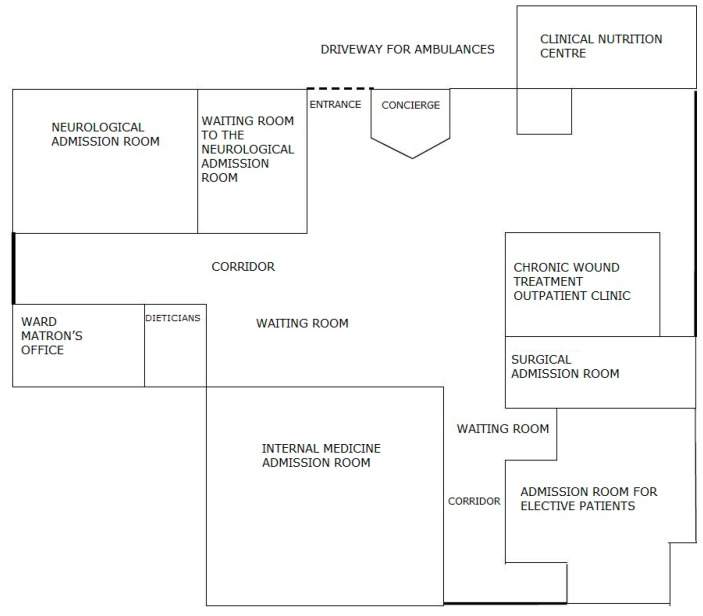
The diagram showing the places in the admission room before the changes were made. Source: own study.

The unit does not have a Hospital Emergency Department (HED); there are admission rooms treating patients depending on the type of disease. There are six structurally separated rooms: internal medicine, gastroenterological, surgical, neurological, and infectious admission room, as well as an admission room for children. There are five admission rooms in the building for adult patients, four of them in the same location, i.e., building A/A1. Within each of the admission rooms, there is a separate room that registers patients for elective procedures. Before the start of the project, elective patients were registered on strictly defined days and hours. The remaining patients, who came with a referral and were brought by ambulance, or those whose health condition required immediate medical assistance, were directed by an employee of the information point to the appropriate room where medical assistance was provided. If the patient had a referral, he/she was referred to the appropriate admission room based on the diagnosis. In a situation where the patient did not have an appropriate document, he/she was referred by a service worker to an internal medicine admission room, or other—if the patient wanted to be seen by a doctor of a specific specialty. Often, while waiting in the corridor for the doctor’s arrival, the patient, initially asked about the ailments by the nurse, was redirected to another area in the admission room. There, he/she was still waiting for a doctor who, after taking medical history and initial examination, could decide to transfer the patient to a doctor of another specialty (based on the initial diagnosis). There were situations when the patient walked/was transported within the admission room from one area to another; each subsequent doctor re-examined the patient and made an independent decision as to further treatment. This caused significant chaos and unnecessarily prolonged the patient’s stay in the admission room. Such a situation was favored by the layout of the rooms, which, in the medical staff, strengthened the feeling of working for the admission room of the department and not the idea of comprehensive patient care. The patient, on the other hand, was forced to visit subsequent rooms, and even wait in several different queues to be admitted by the appropriate doctor, and then to the hospital. The diagram of the rooms is shown in Figure 5.

After admitting the patient to the appropriate admission room, the nurse entered all the necessary data into the IT system and registered him/her in the admission book (made a manual entry) and called the doctor on duty, who, after examining the patient, made a decision on the necessary tests. The examinations in the system were ordered by a nurse who also collected material for tests. The patient waited for the results, which were sent via pneumatic mail from the hospital laboratory. After receiving the test results, the doctor examined the patient once again and made a decision on whether or not to admit the patient to the ward. Any patient who was referred to the hospital ward was transported to the appropriate room under the care of a paramedic. In the department, registration in the system was made again by a nurse. The basic data of the patient, entered into the system in the admission room, also appeared in the ward. However, it was necessary to again complete the paper documentation. Further tests were ordered. Each of the departments and the admission room used different order card templates, which resulted in frequent mistakes when completing them and the need to complete them once again. Very often, it was only at the ward that it was discovered that the documentation collected at the admission room lacked key information—e.g., telephone number of the next of kin and telephone number of the patient. Keeping the patient’s medical records was also significantly difficult because most of the documentation was kept in a paper version. The hospital already had an IT system for the introduction of electronic documentation, but the appropriate forms were not entered into the program, and the medical staff did not have sufficient knowledge regarding the operation of the system.

After treatment, the patient was discharged home by the attending physician. At the ward, he/she waited for the arrival of his/her family, left the hospital on his/her own, or waited for the arrival of the transport ordered by the hospital.

#### 2.3.2. Findings Made during Interviews

As part of the project, standardized interviews were conducted with selected people from the hospital management board and middle-level managers working in the departments participating in the project. The following persons were interviewed: Deputy Director for Treatment, Head of the Department of Internal Medicine, Ward matron of the Department of Gastroenterology, and Deputy Director for Finance. The interviewees were purposefully selected. They were directly responsible for carrying out the project in the hospital: medical issues—Deputy Director for Treatment, costs—Deputy Director for Finance, medical staff—Head of VI Internal Medicine Department, and nursing staff—Ward matron of the VII Department of Gastroenterology. The respondents belonged to the management staff of the Hospital. This is a group of persons with extensive experience in the healthcare sector (from 15 years, in the case of the Deputy Financial Director, to over 20 years of managerial experience, in the case of the remaining respondents). They belonged to the age groups: 45–50 years—two persons—and 55–60 years—two persons. Three of the respondents had a medical degree, while the Deputy Director of Finance had an MBA. At the same time, these were people who had not worked with Lean Management tools before, with the exception of the Deputy Director of Finance, who had been exposed to this management concept in previous jobs.

The director of the hospital and the respondents, who gave their informed consent to participate in the study, consented to the interviews. A qualitative questionnaire was prepared for this purpose. The interview was conducted in the first weeks of the project implementation and after its completion.

The first sheet (Supplementary File S2) contained the following questions:

1. What areas within the Internal Medicine Department, Department of Gastroenterology, and the Admission Room for Adults need improvement?
2. What tasks performed by medical personnel engage them to the greatest extent and are not directly understood as providing health services?
3. What are your expectations towards the implementation of the Lean Management project?
4. Do you have any concerns about the project? What are they regarding?

The next sheet (Supplementary File S3) was part of the interviews conducted after the end of the project; it contained two questions:

1. What problems were solved during the project implementation?
2. Have the concerns you had before starting the project been confirmed? If so, which ones? If not, what had a direct impact on the elimination (reduction) of the concerns?

## 3. Results

### 3.1. Identified Waste

Wastes identified at particular stages of patient admission and treatment were ranked in nine main groups:

1. searching and explaining;
2. waste of overprocessing;
3. waste of defects;
4. waste of overproduction;
5. waste of waiting;
6. waste of motion;
7. waste of human potential;
8. waste of inventory;
9. blame.

Two groups have been added, wasted human potential and blame, which reflect a huge problem in the hospital affecting the effectiveness of the unit and organizational culture.

The ranked wastes are presented in Supplementary File S4.

**Figure 6:**
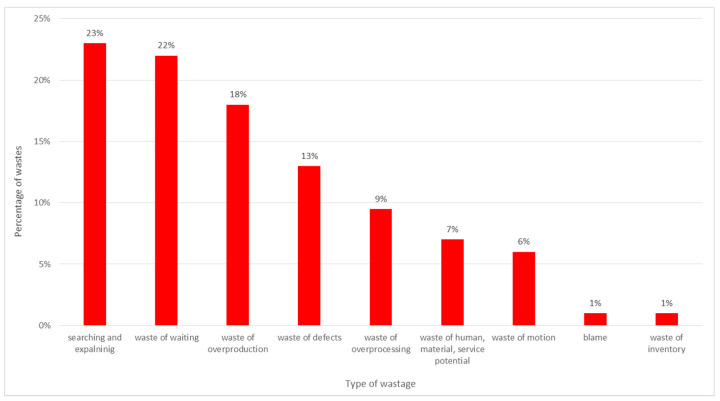
Identified wastes. Source: own study based on the hospital materials.

A percentage distribution of the most common wastes in the unit, broken down into groups, is presented in Figure 6.

The analysis identified a total of 137 different problems. Their distribution into nine groups and a proposal of possible solutions is presented in Table 1.

The most common waste in the unit are activities devoted to searching for information, both information regarding the patient and the information needed to conduct the process of treatment (e.g., telephone numbers at which patient transport can be ordered, key information regarding changes in the principles of operation of laboratories or the method of patient appointments, the lack of guidelines for patient management after diagnostic tests). In addition, the results of observations made in the admission room showed that the medical staff spent a lot of time explaining/informing the patient on issues not related to the process of treatment—for example, about what to take to the hospital, things he/she cannot have during the examination, where to go to collect personal belongings, etc. Additionally, the patients in the room were looking for someone to help them, and they were sent from one room to another. Subsequent doctors performed tests and sent the patient further away. The patient was then awaiting further examination or admission and examination by medical staff.

### 3.2. Changing the Schedule of Admitting a Patient

**Figure 7:**
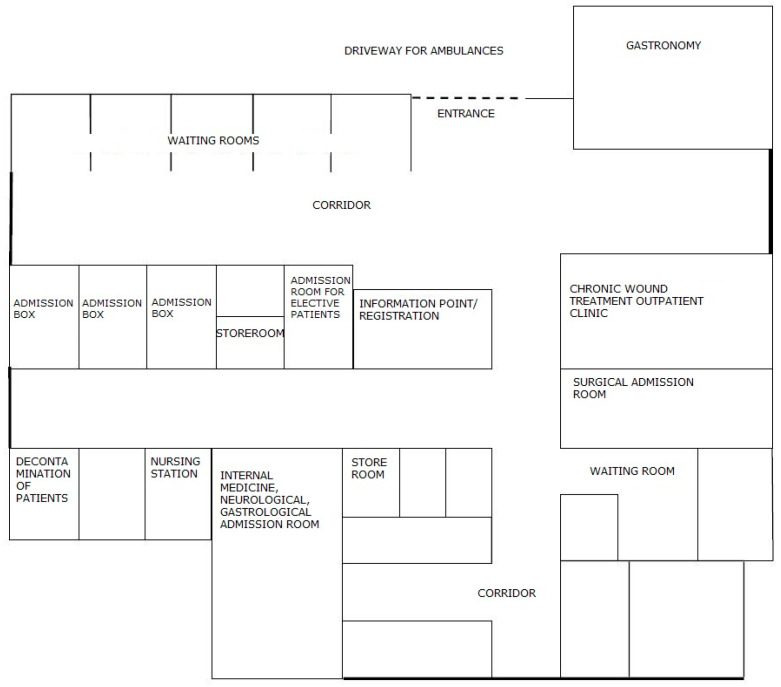
The scheme of the rooms in the admission room after the changes had been made. Source: own study.

After identifying numerous problems in the admission room, the Board of the Hospital decided to rebuild it. Designers, in cooperation with lean experts and with the participation of the employees, redesigned the rooms so that they allowed for the optimal patient flow. The new diagram of places in the admission room is shown in Figure 7.

Architectural changes allowed the hospital to provide the patient with comprehensive care from the moment of entering the admission room. The change in room layout resulted in improved patient flow and direct patient identification and registration. At the same time, it provided greater supervision of patients, improved the efficiency of staff work, and ensured better patient waiting comfort. Now, immediately after entering the hospital, the patient is directed to the centrally located registration. There, his/her data is entered into the system (which relieves the nursing staff from unnecessary activities, who, before the changes, had to register the patient), and then the patient is directed to the admission box. Depending on whether the patient has a referral or presents with urgent ailments, he/she is seen by a specialist based on the diagnosis or by an internist. In the second case, after taking medical history and examining the patient, the physician decides whether to call a specialist in another field for consultation, admit the patient to the ward, or refuse admission if there are no indications for hospitalization.

**Figure 8:**
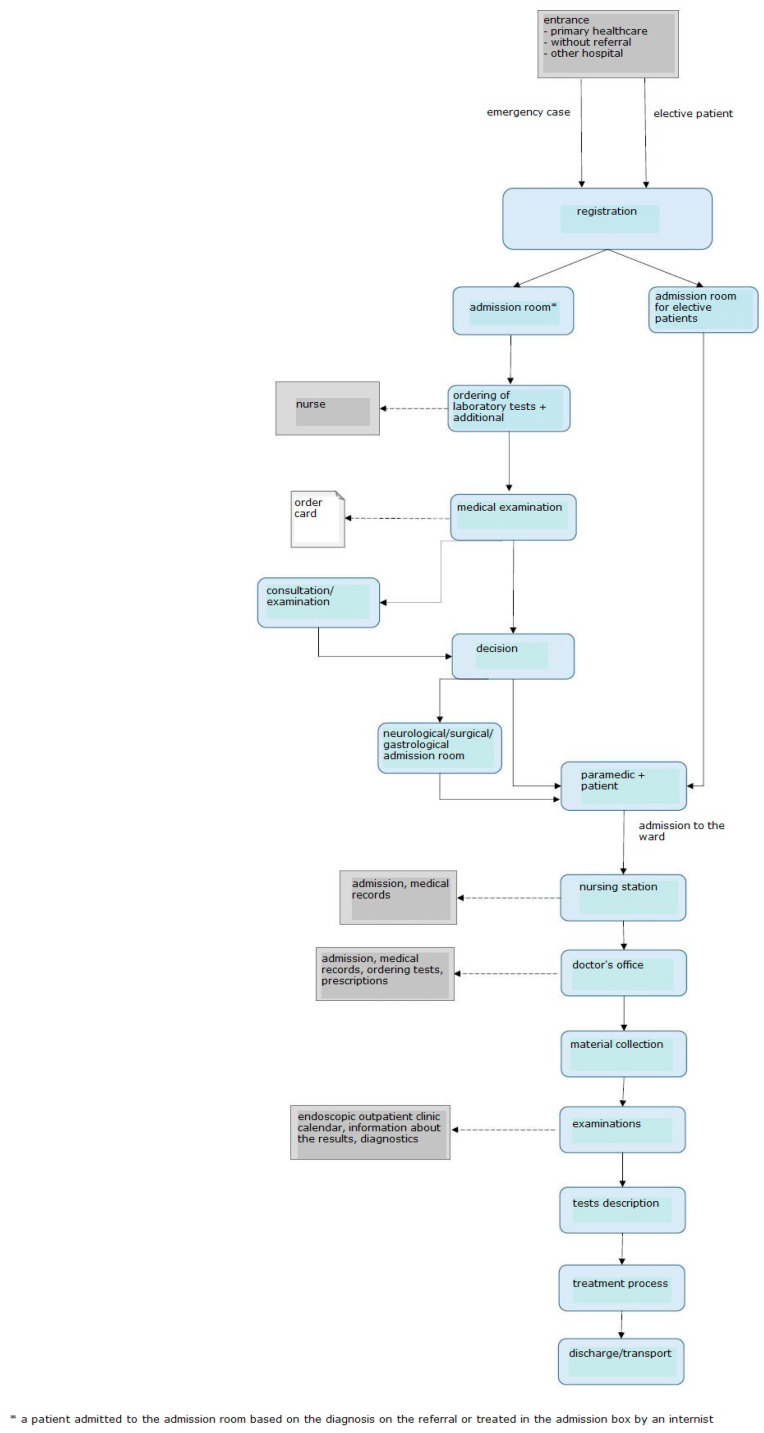
The scheme of admitting the patient after the changes have been made. Source: own study.

The patient no longer has to move from one room to another. He/she is fully cared for in the admission box and then transferred to a given admission room in order to be admitted to the ward. In addition, the new construction solutions provide patients with great comfort, even when waiting for admission. Before the renovation, the patients waited for admission in the corridor. There was no place to ensure privacy and the possibility of peaceful waiting for the examination. There were only a few chairs prepared for these patients in the corridor. Currently, the admission room has a separate waiting room with several separate rooms open to the corridor. This arrangement of the rooms allows for the comfort and intimacy of patients while maintaining safety. Universal admission boxes allow the patient to be examined by a doctor of any specialty, who then decides on the further diagnostic path. The patient is the center of attention of the hospital staff, who can optimally care for him/her and provide necessary assistance. Changing the layout of the rooms forced a change in the way of admitting the patient, which is presented in Figure 8.

Another solution for improving the patient’s admission to hospital, proposed in the field of Visual Management, was the introduction of simple and clear instructions for patients to prepare for a hospital stay. The solution allowed the hospital to not only use the medical staff’s working time more efficiently, but also to reduce the patient’s stress prior to arrival at the hospital. The information—also in an electronic version—was placed on the hospital website.

### 3.3. Solutions for Collection, Processing, and Transmission of Information

Next in order of the number of identified wastes were problems related to overproduction, correction of deficiencies and errors, and overprocessing.

**Figure 9:**
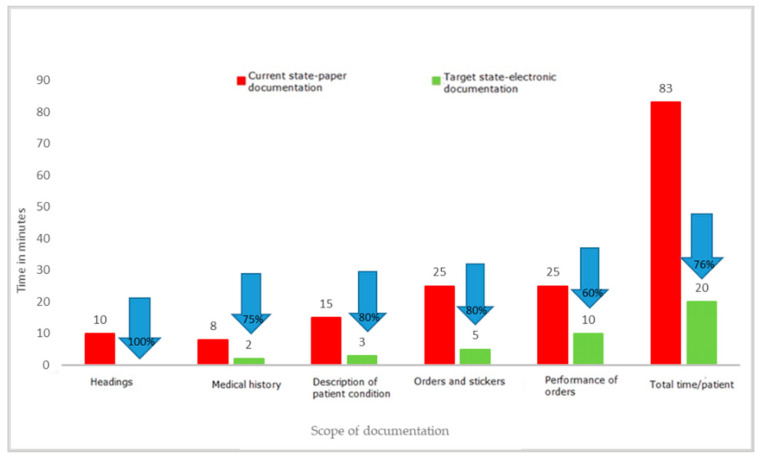
Time to complete the nursing documentation of a newly admitted patient. Source: Own study based on the hospital materials.

**Figure 10:**
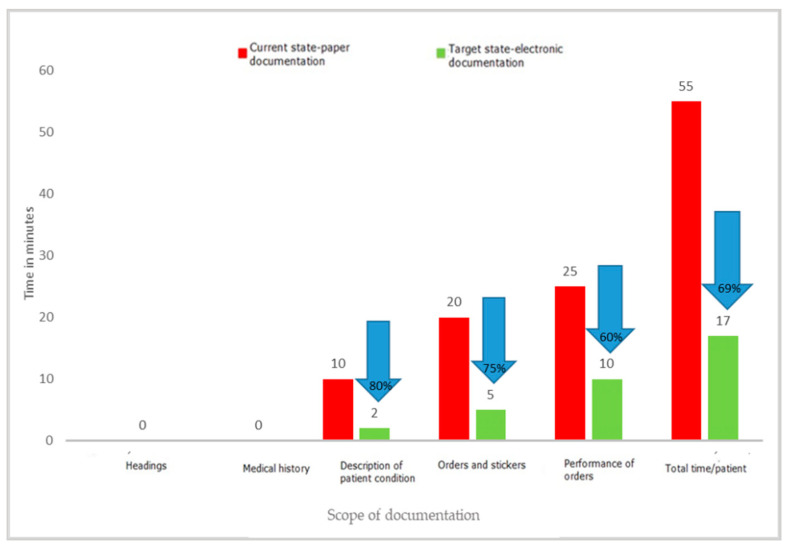
Time to complete the patient’s nursing documentation in the ward. Source: Own study based on the hospital materials.

**Figure 11:**
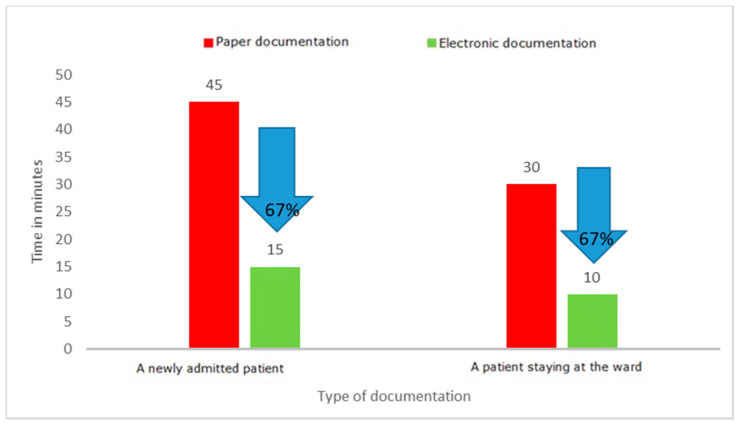
Time to complete the medical documentation. Source: Own study based on the hospital materials.

One of the key activities that temporarily involve medical personnel and do not belong to strictly medical activities is completing medical documentation. It is an activity that must be performed by both doctors and nurses; it is one of their basic duties, but often takes a significant amount of time. During the project, the time needed to complete the patient’s medical documentation and other documents prepared in the wards was measured. The employees of the departments filled in the documentation in the paper version. Measurements were made on the wards using direct observation and time measurements on stopwatches. The individual results of each measurement were recorded on observation sheets and then transferred to an Excel spreadsheet. The experiment with the use of standard forms filled in manually by a doctor and a nurse versus the preparation of the same documents in an electronic version clearly indicated the waste of time when choosing the paper version. Detailed results of the experiment are presented in Figure 9, Figure 10 and Figure 11.

The results of the project clearly show that filling in paper documentation, both for a newly admitted patient and for a patient staying in the ward, is much more time-consuming than filling in the same documentation in an electronic version. The time-savings were, respectively, 63 min for a patient admitted to the hospital and 38 min for an already hospitalized patient. The results may differ depending on the capabilities and competences of the person completing the documentation, which was also confirmed by the experiment, which covered both young people working perfectly in modern IT systems, as well as the elderly. In each case, the electronic version turned out to be faster than the paper version. This was mainly related to the elimination of unnecessary activities, such as entering headings, completing the patient’s data again and again (in subsequent columns), and the need to rewrite the basic results or information regarding the tests ordered. At the same time, the results of the observations revealed that other documents were also kept in paper versions, the filling of which required a significant involvement of medical personnel. These were various types of registers: scheduled admissions, ordered transports, queues for treatments, and imaging examinations.

The analysis of waste identified as excessive processing of paper documents has shown that transferring documents from paper to electronic version brings measurable benefits, such as time savings for medical staff. With an average number of new admissions to the ward of 350 patients per month, the time savings for nursing staff are 368 h (2.3 FTEs) and 175 h (1.09 FTEs) for medical staff. The value of the staff working time saved was EUR 3089/month (14,173.00 PLN/month) for nurses and EUR 2855/month (PLN 13,100.00/month) for specialist doctors. This is one of the key reasons to modify the current ways of working. In the face of large shortages of medical personnel in the health sector, all solutions that allow for more effective management of human capital in the organization are necessary.

### 3.4. The Waiting Time of the Patient for Transport Home

**Figure 12:**
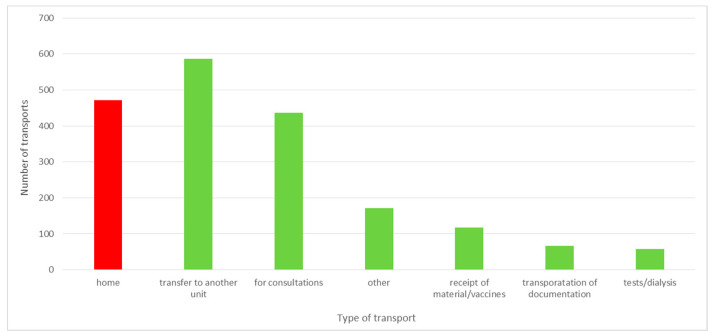
The number of transports ordered by the hospital in the second half of 2019. Source: own study based on hospital data.

As a result of the project, waste related to the patient’s waiting for transport home was identified. The patient was discharged from the ward, awaiting the arrival of his/her family, or left the hospital on his/her own. In the case of 472 patients discharged in the period from 1 July 2019 to 31 December 2019, it was necessary to transport them by external hospital transport, which repeatedly picked them up with a significant delay. This limited the possibility of admitting a new patient to the ward. The analysis of patient transports home ordered by the hospital showed that over 10% of the transports took place after 8 p.m. and almost 6% after 10 p.m. Pursuant to the Act on healthcare services financed from public funds [31], a patient with a motor organ dysfunction, which prevents him/her from using public transport, is entitled to sanitary transport provided by a medical entity free of charge on the basis of a doctor’s order. If the patient can walk, sanitary transport is available for a fee or a partial payment. The analysis of the transports carried out in the second half of 2019 revealed that patient transports home constituted 25% of all the transports. A distribution of types of transports ordered by the hospital is presented in Figure 12.

Of all patient transports home, more than 4% were ordered for walking patients for whom, in accordance with the provisions of the above-mentioned Act, the hospital is not obliged to provide transport or may, in return, demand reimbursement of funds. According to the information obtained from the Finance and Accounting Department, the hospital did not invoice patients’ home transports. Each time the costs of the transport were borne by the hospital. The transport of walking patients home was most often ordered by the staff of the Neurological Admission Room. The direct observations revealed that this was due to the staff’s fear of the legal consequences of a possible event on the patient’s way home.

Moreover, the analysis showed that 60% of the transports were ordered between 3 p.m. and 4 p.m. This could be the reason for waiting so long for an ambulance. In 3% of cases, the ambulance came to pick up the patient more than 10 h after the transport order (these were usually night hours, e.g., 1:00 a.m., 2:30 a.m.). This resulted in the necessity to extend the stay of the patient already discharged from the hospital in the ward. In several situations, the doctor decided not to transport the patient home at night for the good of the patient.

Another large group of orders were transports of patients for consultations to another hospital—23%, or transfers of patients to other entities—31%. At the same time, 14% of transports were transports of medical documentation for the purpose of external consultations. This transport could be replaced by introducing teleinformatic consultations. The hospital has a sufficient technical infrastructure. It would only be necessary to conclude agreements with appropriate units and agree on the means of communication through which the patient’s documents would be safely accessible.

### 3.5. Analysis of Prolonged Stays in Wards

Prolonged stays are another waste identified in the project. One of the reasons for them was the inability to take the patient home (no immediate family to take care of the patient—so-called stays for social reasons). Direct observations showed that this situation usually concerned the elderly, sick, and those requiring constant care. These patients remained in the hospital ward until someone from their family came for them, or a hospital social worker found a place for such a person in a care and treatment facility. Waiting for the end of hospitalization also concerned situations in which the patient could not be discharged because the necessary tests or consultations had not been performed, or their results were not yet available. This is a big challenge, not only in terms of organization; prolonged stays of patients in hospital wards increase the risk of nosocomial infections. They are also a general measure of hospital efficiency and are directly related to cost reduction [32].

The aforementioned waste requires a broader analysis of the causes and the application of organizational, legal, and financial solutions to shorten the patient’s stay in the hospital and, subsequently, reduce the risk of recurrent nosocomial infections.

### 3.6. Discussion of Interview Results

Some very important elements considered before the implementation of the project were communication problems reported by the staff (the lack of communication or inadequate communication within the organization), errors in the medical documentation, and the lack of time to correct the mistakes along with the need to complete the current documentation, as well as problems reported by patients, such as the lack of necessary information, long waits to be admitted to hospital, and no precise instructions. The attitude of the medical staff towards new challenges was another major challenge. Despite the problems reported by the staff, there was resistance and reluctance to change habits. This was often accompanied by the fear of the unknown and of using new technologies at work (especially in the case of older doctors). In the first phase of the project, the Deputy Medical Director especially expressed his distrustful attitude; his doubts were consistent with those expressed by the medical and nursing staff.

Interviews with persons responsible for the implementation of the project highlighted the concerns of individual groups of staff related to the implementation of new solutions in the healthcare unit. There were doubts as to the possibility of observing the effects in a short period of time. This fear led to discouragement and distrust of the project. In the opinion of the interview participants, it could also lead to the lack of involvement of line employees and middle-level managers in the departments. Moreover, fears related to the improvement of individual processes and activities of organizational units were expressed. Improving the work of one of the departments could, in the opinion of the staff, lead to a deterioration in functioning of another. The interviews revealed organizational problems with ordering examinations, carrying out transports, transferring patients to other units, and performing consultations, as well as with completing paper documentation. There were also problems with cooperation and communication between organizational units.

The interviews confirmed the silo approach of the departments and the fact that they worked exclusively for their own good without taking into account the hospital as a whole. The expectations regarding the implementation of the project concerned primarily the improvement of the organization of work inside the hospital and easier completion of documentation, as well as the improvement of the IT system, but also changes in the organizational culture—focusing on the patient as a customer of services. Moreover, the need to introduce changes in personnel management was formulated. In terms of rationalization of employment in individual departments, the following were taken into account: the current needs of the hospital, the epidemiological situation, and occupancy of the department, as well as building commitment to work for the benefit of the patient and the hospital and improving work efficiency by linking the effects with measurable financial effects for the employee.

The interviews conducted after the implementation of Lean Management tools showed that the project did not meet all expectations. The respondents indicated a noticeable improvement in filling in medical documentation, which allowed for the acceptance of new solutions by all medical workers in the departments covered by the project. The employees positively responded to the effects of the improvements; however, some of them expected spectacular changes in a short period of time. The high complexity and fragmentation of the organization made the operation of the hospital inflexible, and this was noticed by the interviewees. Moreover, they drew attention to the significant influence of the external factors (regulators—the Ministry of Health, the National Health Fund and supervisory bodies), which prevent the implementation of dynamic changes. Top-down regulations hinder the introduction of an incentive system for hospital employees, e.g., regulations changing the rules of remunerating employees without taking into account the specificity of units or their financial resources for pay raises, no possibility of remuneration for the effect of treatment due to the method of valuation of services, or limited supply for the medical worker market, resulting in the need to maintain employment regardless of the results of work—especially in relation to nursing staff for whom statutory employment standards apply [33]. However, the interviews revealed that the introduction of Lean Management tools resulted in the involvement of staff in the implementation of individual solutions; at the same time, noticeable effects and proposed organizational solutions aroused an interest in continuing the implementation of the project in other areas of hospital operations. Moreover, it transpired that the Lean Management training, precisely defined goals, and detailed division of work in groups were of great importance for the positive reception of the project.

## 4. Discussion

The existing implementations of Lean Management in healthcare units around the world show many benefits related to the elimination of waste, such as reduced patient waiting time, reduced number of patient visits, fewer errors, and improved patient and staff satisfaction, as well as increased work efficiency [16,21,34,35,36,37,38]. In the hospital in Huston, the implementation of lean tools in HEDs significantly reduced the patient’s waiting time for assistance, and thus shortened the ED stay [39]. Another study shows a correlation between the overcrowding of emergency medical units and the number of errors made by medical and nursing staff [40]. The implementation of lean tools at Odense University Hospital confirmed the benefits and improvements throughout the organization, in particular in terms of logistics and distribution [2,41]. Additionally, in many places, an improvement in the organization of work and an increase in efficiency of particular organizations were directly observed [25,42].

The most important waste identified after the implementation of LM tools related to the collection, processing, and transfer of information. The introduction of electronic medical documentation in the hospital allowed a shortening of the time of completing it by doctors by 67% and by nurses by 76% for a newly admitted patient, and by 69% for a patient staying in the ward. This is a very important result as it justifies the efforts to broadly computerize the healthcare sector. Starting from 2021, keeping electronic medical records is an obligation imposed by the Regulation of the Minister of Health of 6 April 2020 on types, scope, and templates of medical documentation [43]. The results of the research carried out in the hospital in Wroclaw confirm that computerization of hospitals and electronic medical documentation are the right course of action. However, not all hospitals in Poland are prepared to implement the above obligation [44]. The Minister of Health envisaged solutions enabling adaptation to the new requirements, at the same time, constant digitization of healthcare in Poland is to be implemented [45]. From the very beginning, the process has raised concerns in the medical community, which was confirmed by a survey conducted by the Supreme Medical Chamber and the resultant statement [46].

The project confirmed the claim in the literature that the implementation of Lean Management can improve the functioning of healthcare units by identifying wastes in processes [47]. The use of value-stream mapping allowed for the transformation of the process of admitting the patient to the hospital and treatment, as well as for the identification of defective points. At the same time, the study showed that the attitudes of employees are of great importance when implementing Lean Management. The first fear, reluctance to apply new solutions, may be a factor that will significantly affect the possibility of implementation. However, as the interviews revealed, after the completion of the project and the first positive results, reluctance may turn into openness and interest in new areas and opportunities for improvement. It is important to combine the implementation of Lean Management tools with the elements of work psychology in order to stimulate motivation among employees [48]. An indispensable element of lean implementation is the training and involvement of all hospital workers in improving the inner processes in conjunction with the active support of leaders (through supporting delegation of tasks, joint decision-making, and joint management) [49]. Identifying the waste of processing too much data in the paper version and the involvement of staff in the implementation of electronic documentation meant that positive results were obtained, along with a change in the attitudes of employees as they became more open to IT systems. In the Netherlands, the systems for exchanging data between healthcare facilities via secure e-mails, a regional IT structure, and a national service point, where professionals can share medical information, are increasingly used. After the patient gives his/her consent, the physician has access to his/her data provided at the point by another unit [49].

Research indicates that the problems with the implementation of LM tools include the complexity of processes in hospitals, difficulties in involving medical personnel, and the need to adapt the tools to the specificity of the unit and supplement them with additional methods and instruments available in other management systems [2,41]. According to the study conducted in the Wroclaw hospital, the resistance of employees and the fear of having to learn about the new electronic system may be another problem with the implementation of LM tools. Observations and conversations with employees in the wards confirmed that many of them do not know the system or all of its functions and potential possibilities. The solution was proposed to record tutorials showing how to work in the system and to familiarize employees with new technologies in a way that reduces tension through systematic training, face-to-face meetings, and supporting mutual aid initiatives. Moreover, such IT projects should be planned in great detail, taking into account both the necessary implementation time and the involvement of individual units. In subsequent stages, it will be necessary to monitor the effects to ensure staff involvement and effective implementation [50].

In addition, other wastes were identified during the project that make the work of medical staff significantly more difficult. The analysis revealed potential causes of some difficulties; as a result, possible solutions were presented. The analysis of individual areas of the hospital operation has shown that many of them require further detailed research and improvement. The use of Lean Management tools can be a solution to improve the work and treatment of the patient. At the same time, it is necessary to pay attention to the analysis of the patient’s and family’s satisfaction at each stage of making improvements [51]. This element was not included in the project; therefore, the unit could not evaluate the activities undertaken in this area. Moreover, Lean Management tools were implemented in only three departments. The remaining departments were not covered by the project, which makes it impossible to assess the impact of the implementation on the entire unit. The duration of the project was also a significant limitation, which was strictly defined (1 July 2019–31 December 2019). The hospital management decided to continue, in 2020, cooperation with external experts and thus expand the area of implementing LM tools throughout the hospital. Unfortunately, the outbreak of the SARS-CoV-2 pandemic made it impossible to implement the project in its original form. The hospital was entirely dedicated to providing services to patients infected with the virus, therefore the participation of experts and the implementation of the project were focused on preparing the hospital to fight the pandemic.

## 5. Conclusions

The use of Lean Management tools in the hospital allowed for identification of the waste in the process of admitting and treating a patient. Defining the problems and implementing improvements allowed for the development of new work standards that significantly shortened the time for staff to perform activities not directly related to the treatment of the patient. Working in teams resulted in the involvement of not only managers, but also the staff working directly with the patient. It was one of the key elements of the project’s success that allowed for the reduction of resistance to new solutions.

The project confirmed the possibility of implementing the principles of Lean Management in the healthcare sector in Poland. The situation of Polish hospitals is currently very difficult. They are struggling with financial problems resulting from the under-financing of the healthcare sector in Poland and the shortage of human resources among medical personnel. In 2019, Poland spent 6.5% of its GDP on healthcare expenditure, which places it in 26th place out of 31 EU countries [52]. In addition, in 2018, in the Euro Health Consumer Index report, Poland was ranked 32nd out of 35 countries in Europe, achieving one of the worst results in all areas, especially in terms of patient rights, information, availability, and range and scope of operation [23].

The use of Lean Management in the hospital presented in this article allowed for a significant improvement in the efficiency of the work of medical personnel and for significant savings in working time that could be spent on patient care. The implemented solution resulted in a better use of the human capital resources available in the unit, which is one of the key challenges faced by healthcare sector units in Poland. The deteriorating staff situation, visible in Figure 1 and Figure 2, necessitates the use of solutions that will eliminate unnecessary activities performed by medical personnel, so that a limited number of people can efficiently perform basic tasks related to the provision of health services.

The experiences of other countries show that the use of Lean Management in healthcare has positive effects, and it is a desired direction of hospital development. The method taken from the manufacturing sector brings real benefits, for instance, increased efficiency and savings [53]. Under the conditions of the large financial and staffing problems of hospitals, the use of Lean Management in Poland seems to be a necessity, while the number of LM implementations both in the Polish healthcare sector and in units in the eastern part of Europe is still small [22,30]. Thanks to the research and the results presented in the article, all organizations interested in Lean Management will be able to follow the example of the Wroclaw hospital, and thus they will implement Lean Management tools much faster.

The study and the results presented in the article indicate that, even in a large multispecialty hospital, where the staff had no knowledge or skills regarding how to use new methods of management, and also in the conditions of dynamic changes in the environment caused by the SARS-CoV-2 virus pandemic, it is possible to implement LM solutions. The study, in the form of a case study, also showed that the use of LM tools results, not only in the implementation of many improvements that facilitate the work of medical staff and streamlined the process of providing health services, but also in the improvement of economic efficiency and productivity. This is an important aspect in terms of science, as it points to possible practical solutions to organizational and cost-effectiveness problems that arise in hospitals. The example of this study should help increase interest in the concept of Lean Management in healthcare system. The operationalization of particular elements of LM concept implementation and the effectiveness of introduced changes, presented in the article, can be an inspiration and also a model for carrying out a similar process in other units.

## Figures and Tables

**Table 1:** A distribution of wastes and proposed solutions by groups.

Type of Waste	Number of Identified Wastes	Number of Proposed Solutions
searching and explaining	31	21
waste of waiting	30	22
waste of overproduction	25	19
waste of defects	18	17
waste of overprocessing	12	11
waste of human, material, service potential	10	6
waste of motion	8	7
blame	2	0
waste of inventory	1	1
**Total**	**137**	**104**

## Data Availability

The data presented in this study are available on request from the corresponding author.

## Supplementary Materials

The following supporting information can be downloaded at: https://www.mdpi.com/article/10.3390/ijerph19020800/s1, File S1. The A3 report. File S2. Pre-project interview sheet. File S3. Post-project interview sheet. File S4. Waste identified in the project.

## References

[1] Aoun M., Hasnan N., Al Aaraj H. (2018). Relationship between Lean Practices, Soft Total Quality Management and Innovation Skills in Lebanese Hospitals. East. Mediterr. Health J..

[2] Kovacevic M., Jovicic M., Djapan M., Zivanovic-Macuzic I. (2016). Lean Thinking in Healthcare: Review of Implementation Results. Int. J. Qual. Res..

[3] Graban M., Kubik S. (2011). Lean Hospitals—Improving Hospitals: Improving Quality, Patient Safety and Staff Satisfaction.

[4] Jakonis A., Difin (2017). Cultural Determinants of Lean Management.

[5] Buczacki A., Gładysz B., Timler D. (2019). Industrial Engineering for Healthcare Management—Example Lean Management and ICT Tools. Stud. Log. Gramm. Rhetor..

[6] Stockfisch V. (2011). Lean Management in Hospitals: Principles and Key Factors for Successful Implementation.

[7] Heinbuch S.E. (1995). A Case of Successful Technology Transfer to Health Care: Total Quality Materials Management and Just-in-time. J. Manag. Med..

[8] Young T.P., McClean S.I. (2008). A Critical Look at Lean Thinking in Healthcare. Qual. Saf. Health Care.

[9] Ferreira W.D.P., Da Silva A.M., Tanaka W.Y., Zampini E.D.F. (2016). Lean & Healthcare Organizations—A Systematic Literature Review with Bibliometric Analysis on Application of Lean Healthcare in Brazil. Braz. J. Oper. Prod. Manag..

[10] Brandao de Souza L. (2009). Trends and Approaches in Lean Healthcare. Leadersh. Health Serv..

[11] Radnor Z.J., Holweg M., Waring J. (2012). Lean in Healthcare: The Unfilled Promise?. Soc. Sci. Med..

[12] Pfannstiel M.A., Rasche C. (2017). Service Business Model Innovation in Healthcare and Hospital Management: Models, Strategies, Tools.

[13] Edwards K., Nielsen A.P. Improving Healthcare through Lean Management: Experiences from the Danish Healthcare System. Proceedings of the 5th Nordic Conference on Health Organization and Management.

[14] Ōno T. (1988). Toyota Production System: Beyond Large-Scale Production.

[15] Alnajem M., Garza-Reyes J.A., Antony J. (2019). Lean Readiness within Emergency Departments: A Conceptual Framework. Benchmarking Int. J..

[16] de Souza L.B., Pidd M. (2011). Exploring the Barriers to Lean Health Care Implementation. Public Money Manag..

[17] Decker W.W., Stead L.G. (2008). Application of Lean Thinking in Health Care: A Role in Emergency Departments Globally. Int. J. Emerg. Med..

[18] Chiarini A., Baccarani C. (2016). TQM and Lean Strategy Deployment in Italian Hospitals: Benefits Related to Patient Satisfaction and Encountered Pitfalls. Leadersh. Health Serv..

[19] Poksinska B. (2010). The Current State of Lean Implementation in Health Care: Literature Review. Qual. Manag. Health Care.

[20] Drotz E., Poksinska B. (2014). Lean in Healthcare from Employees’ Perspectives. J. Health Organ. Manag..

[21] Dickson E.W., Singh S., Cheung D.S., Wyatt C.C., Nugent A.S. (2009). Application of Lean Manufacturing Techniques in the Emergency Department. J. Emerg. Med..

[22] Korkosz-Gębska J., Gębski J. Standardy LeanOZ. Mapping of Value Streams in Health Care 2018. https://leanoz.pl/uploads/pdev_ftd/Standardy%20LeanOZ-2.pdf.

[23] Health Consumer Powerhouse (2019). Euro Health Consumer Index. 2018.

[24] (2020). Health and Health Protection in 2019. Report of the Central Statistical Office. https://stat.gov.pl/obszary-tematyczne/zdrowie/zdrowie/zdrowie-i-ochrona-zdrowia-w-2019-roku,1,10.html.

[25] Dannapfel P., Poksinska B., Thomas K. (2014). Dissemination Strategy for Lean Thinking in Health Care. Int. J. Health Care Qual. Assur..

[26] Brajer-Marczak R., Wiendlocha A. (2018). Lean Management Concept in Hospital Management—Possibilities and Limitations. Manag. Sci..

[27] Womack J.P., Jones D.T. (2003). Lean Thinking: Banish Waste and Create Wealth in Your Corporation.

[28] Bhasin S. (2015). Lean Management beyond Manufacturing.

[29] Narayanamurthy G., Gurumurthy A. (2018). Is the Hospital Lean? A Mathematical Model for Assessing the Implementation of Lean Thinking in Healthcare Institutions. Oper. Res. Health Care.

[30] Preś I., Dudek M., Motowidlak U., Wronkowski D., Reńda A. (2018). Rozwiązania Lean Management w placówkach ochrony zdrowia na świecie. Case study: Wdrożenie metod Lean Management w Regionalnym Centrum Nefrologii w Szczecinku. Różne Oblicza Logistyki. Zbiór Prac Studentów.

[31] Act of Law of 27 August 2004 on Health Care Benefits Financed from Public. https://isap.sejm.gov.pl/isap.nsf/download.xsp/WDU20042102135/U/D20042135Lj.pdf.

[32] Zepeda-Lugo C., Tlapa D., Baez-Lopez Y., Limon-Romero J., Ontiveros S., Perez-Sanchez A., Tortorella G. (2020). Assessing the Impact of Lean Healthcare on Inpatient Care: A Systematic Review. Int. J. Environ. Res. Public. Health.

[33] Regulation of the Minister of Health of 28 December 2012 on the Method of Determining Minimum Employment Standards for Nurses and Midwives at Non-Enterprise Medical Entities. https://isap.sejm.gov.pl/isap.nsf/download.xsp/WDU20120001545/O/D20121545.pdf.

[34] Toussaint J. (2009). Writing the New Playbook for U.S. Health Care: Lessons from Wisconsin. Health Aff..

[35] Toussaint J.S., Berry L.L. (2013). The Promise of Lean in Health Care. Mayo Clin. Proc..

[36] Smith G., Poteat-Godwin A., Harrison L.M., Randolph G.D. (2012). Applying Lean Principles and Kaizen Rapid Improvement Events in Public Health Practice. J. Public Health Manag. Pract..

[37] Bushell S., Shelest B. (2002). Discovering Lean Thinking at Progressive Healthcare. J. Qual. Particip..

[38] Lawal A.K., Rotter T., Kinsman L., Sari N., Harrison L., Jeffery C., Kutz M., Khan M.F., Flynn R. (2014). Lean Management in Health Care: Definition, Concepts, Methodology and Effects Reported (Systematic Review Protocol). Syst. Rev..

[39] Eller A. (2009). Rapid Assessment and Disposition: Applying LEAN in the Emergency Department. J. Healthc. Qual..

[40] Kulstad E.B., Sikka R., Sweis R.T., Kelley K.M., Rzechula K.H. (2010). ED Overcrowding Is Associated with an Increased Frequency of Medication Errors. Am. J. Emerg. Med..

[41] Hasle P., Nielsen A.P., Edwards K. (2016). Application of Lean Manufacturing in Hospitals-the Need to Consider Maturity, Complexity, and the Value Concept: Application of Lean Manufacturing in Hospitals. Hum. Factors Ergon. Manuf. Serv. Ind..

[42] Sloan T., Fitzgerald A., Hayes K.J., Radnor Z., Sohal S.R.A. (2014). Lean in Healthcare—History and Recent Developments. J. Health Organ. Manag..

[43] Regulation of the Minister of Health of 6 April 2020 on Types, Scope and Models of Medical Documentation. http://isap.sejm.gov.pl/isap.nsf/download.xsp/WDU20200000666/O/D20200666.pdf.

[44] https://www.prawo.pl/zdrowie/dokumentacja-medyczna-od-1-stycznia-2021-roku-co-elektronicznie,505362.html.

[45] https://www.nfzszczecin.pl/swiadczeniodawcy_news_3589_e_dokumentacja_medyczna_jak_przebiega_wymiana_elektronicznej_dokumentacji_medycznej_miedzy_placowkami.htm?phpsessid=5ddd0801c1333ef1e0076229cbb399af.

[46] https://www.medexpress.pl/samorzad-lekarski-w-sprawie-obowiazkow-zwiazanych-z-elektroniczna-dokumentacja-medyczna/81809.

[47] Cardoso W. (2020). Value Stream Mapping as Lean Healthcare’s Tool to See Wastage and Improvement Points: The Case of the Emergency Care Management of a University Hospital. Rev. Gestão Sist. Saúde.

[48] Hopp W.J. (2018). Positive Lean: Merging the Science of Efficiency with the Psychology of Work. Int. J. Prod. Res..

[49] Clancy G., Graban M. (2014). Engaging Staff as Problem Solvers Leads to Continuous Improvement at Allina Health. Glob. Bus. Organ. Excell..

[50] Harrison M.I., Paez K., Carman K.L., Stephens J., Smeeding L., Devers K.J., Garfinkel S. (2016). Effects of Organizational Context on Lean Implementation in Five Hospital Systems. Health Care Manag. Rev..

[51] Poksinska B.B., Fialkowska-Filipek M., Engström J. (2017). Does Lean Healthcare Improve Patient Satisfaction? A Mixed-Method Investigation into Primary Care. BMJ Qual. Saf..

[52] OECD (2017). European Observatory on Health Systems and Policies. Polska: Profil Systemu Ochrony Zdrowia 2017.

[53] Horbal R., Kagan R., Koch T., Sobczyk T. Minione 10 Lat Ruchu Lean w Polsce. Wnioski i Perspektywy [Last 10 Years of Lean in Poland. Conclusions and Perspectives]. https://lean.org.pl/minione-10-lat-ruchu-lean-w-polsce-wnioski-i-perspektywy/.

