# Supplementary material for: Implementation of Lean Management in a Multi-Specialist Hospital in Poland and the Analysis of Waste
Source: Int J Environ Res Public Health. 2022 Jan 12;19(2):800. doi: 10.3390/ijerph19020800 (PMC8775623; doi:10.3390/ijerph19020800)
Supplement: Supplementary file 1 [file ijerph-19-00800-s001.zip › File S1 - A3 Report.pdf]

**DEPARTMENT:**<br>**PROCES:**	**REPORT A3**	**DATE:** **LEADER:**<br>**PARTICIPANTS:**

1) DEFINITION OF THE PROBLEM	5) DEVELOPMENT OF REMEDIAL MEASURES
2) ANALYSIS OF THE PROBLEM	6) IMPLEMENTATION<br>**DO**
**PLAN**<br>3. SETTING A TARGET	7) VERIFICATION<br>**CHECK**
4) SEARCHING FOR THE CAUSES OF THE PROBLEM	8) STANDARDISATION<br>**ACT**
