# Supplementary material for: Implementation of Lean Management in a Multi-Specialist Hospital in Poland and the Analysis of Waste
Source: Int J Environ Res Public Health. 2022 Jan 12;19(2):800. doi: 10.3390/ijerph19020800 (PMC8775623; doi:10.3390/ijerph19020800)
Supplement: Supplementary file 1 [file ijerph-19-00800-s001.zip › File S2 - Pre-project interview sheet.pdf]

# A.2

| Question | Answers |
| --- | --- |
| | Interviewee： |
| What areas in the Internal Medicine Department, Gastroenterology Department and Admission Room for Adults need improvement? | |
| What tasks performed by medical personnel engage them to the greatest extent and are not directly understood as providing health services? | |
| What are your expectations towards the implementation of a lean management project? | |
| Do you have any concerns about the project? What are they about? | |
