# Supplementary material for: Implementation of Lean Management in a Multi-Specialist Hospital in Poland and the Analysis of Waste
Source: Int J Environ Res Public Health. 2022 Jan 12;19(2):800. doi: 10.3390/ijerph19020800 (PMC8775623; doi:10.3390/ijerph19020800)
Supplement: Supplementary file 1 [file ijerph-19-00800-s001.zip › File S3 - Post-project interview sheet.pdf]

A.3

Question	Answers
	**Interviewee:**
Have the project met your expectations? How do you evaluate the way it was carried out and the proposed solutions?	
Have the concerns you had before starting the project been confirmed? If so, which ones and why?	
